# The role of adipose triglyceride lipase in lipid and glucose homeostasis: lessons from transgenic mice

**DOI:** 10.1186/s12944-019-1151-z

**Published:** 2019-11-22

**Authors:** Michael J. Trites, Robin D. Clugston

**Affiliations:** 1 Department of Physiology, Faculty of Medicine and Dentistry, University of Alberta, Edmonton, Alberta T6G 2H7 Canada; 2 Group on the Molecular and Cell Biology of Lipids, Faculty of Medicine and Dentistry, University of Alberta, Edmonton, Alberta Canada

**Keywords:** Adipose triglyceride lipase (ATGL), Glucose metabolism, Lipid metabolism, Transgenic mice, Triglyceride (TG)

## Abstract

The ability of mammals to store and draw on fat reserves has been a driving force throughout evolution in an environment with intermittent nutrient availability. The discovery of adipose triglyceride lipase (ATGL) as a triglyceride lipase provided a heightened understanding of the mechanisms governing mobilization of fat reserves from adipose tissue. ATGL catalyses the initial step in adipose triglyceride lipolysis, working in concert with other enzymes to mobilize triglyceride for energy production. In addition to the role of ATGL in adipose tissue triglyceride mobilization, ATGL plays crucial roles in regulating lipid homeostasis in other tissues. These roles have been characterized primarily using transgenic mice with tissue-specific ATGL ablation. For example, the global ATGL knockout induces a severe cardiac defect that results in premature mortality that is mimicked by inducible cardiomyocyte-specific ATGL knockout. Global- and adipose-specific ATGL ablation induces a whole-body shift from lipid metabolism to glucose metabolism to satisfy metabolic demand primarily facilitated by an increase in glucose uptake by skeletal muscle. Generation of liver-specific ATGL knockouts has implicated hepatic lipolysis as a critical component of normal liver function. Analysis of β-cell ATGL knockouts implicates the necessity of pancreatic ATGL in insulin secretion. The objective of this review is to discuss the contributions of ATGL to systemic lipid- and glucose-homeostasis discovered through the study of transgenic mice.

## Introduction

Efficient fat metabolism is paramount to maintenance of systemic nutritional homeostasis. Variations in environmental nutrient availability, as well as variations in nutrient intake throughout the day (even in environments with nutrient abundance), necessitate systems of energy storage and liberation to satisfy metabolic demands. Circulating glucose and amino acids, as well as stored glycogen are a source for rapid ATP production in humans and other mammals, but constitute a relatively small pool of energy substrates with glucose and glycogen stores totalling approximately 1500 available kilocalories, compared to fat stored in adipose tissue, which can total approximately 140,000 kcal in a 70 kg adult male [1, 2]. Thus, successful transition from a fed to fasted state requires mobilization of fat stores. In a typical adult, lipids comprise 80% of energy reserves. Although these lipid reserves are primarily found as triglycerides (TG) stored in adipose tissue, most tissues store at least some TG in lipid droplets [2, 3]. Subsequent mobilization of energy stores by TG lipolysis during fasting is therefore an important metabolic process in cells throughout the body.

The discovery of ATGL as an intracellular lipase has been key not only to understanding lipolysis regulation in multiple tissues, but also the integration between glucose and lipid metabolism as primary substrates for energy metabolism. While the role of ATGL in lipolysis and its association with hormone-sensitive lipase (HSL) and the coregulators CGI-58 and G0S2 has been previously reviewed [4–7], here we will uniquely focus on the generation of transgenic mice with global- and tissue-specific ablation of ATGL that have led to advances in our knowledge and understanding of ATGL’s contribution to integrative energy homeostasis.

With regards to nomenclature, the discovery of ATGL was unique in that several groups independently identified and characterized this enzyme, and hence in the earlier literature the protein was variably referred to as ATGL, desnutrin, PNPLA2, or calcium independent phospholipase A_2_ (iPLA_2_), while the gene has been referred to as *Pnpla2* or *Tts2.2* [8–11]. In this review, the nomenclature ATGL and *Pnpla2* will be used for protein and gene, respectively. In terms of gene and protein structure, the *Pnpla2* gene in mice is found on chromosome 7 and is approximately 6 kb. The gene contains nine exons and the mRNA is approximately 2 kb. The murine ATGL protein is composed of 486 amino acids and is approximately 54 kDa. ATGL has highly sequence homology with other members of the patatin-like phospholipase domain proteins PNPLA1, PNPLA3, PNPLA4, and PNPLA5 [12].

## Glucose metabolism is improved in global ATGL deficient mice

For almost three decades HSL was considered to be the predominant intracellular TG lipase in adipose and other tissues, as recently reviewed [5, 6]. However, HSL-deficient mice did not accumulate adipose TG, but instead accumulated diglyceride (DG), and were resistant to diet-induced obesity [13, 14]. This unexpected finding catalysed the search for, and discovery of ATGL as the predominant adipose TG lipase [5, 6].

The role of ATGL in lipolysis of TG was initially believed to be adipose-specific, since only minute protein and mRNA expression was detected in other tissues such as skeletal muscle, cardiac muscle, kidney and testis of male mice [11]. Shortly after those initial reports, however, ATGL-deficient (*Pnpla2*^*−/−*^) mice were generated [15]. As expected, they displayed striking contrasts to HSL-deficient mice, and maintained adipose TG levels approximately 2-fold higher than wild type controls [13, 15]. Unexpectedly, however, they also accumulated a significant excess of TG in all tissues tested, including a 20-fold increase in cardiac muscle, 15-fold increase in kidney, 4-fold increase in pancreas, 3-fold increase in skeletal muscle, and a 2-fold increase in liver [15]. This suggested important tissue-specific roles for ATGL beyond adipose tissue, and a series of tissue-specific *Pnpla2*^*−/−*^ mice have now been generated to investigate these unexpected findings. Contrasting those tissue-specific models with findings from the global knockout model provide significant insight into the systemic and local integration of TG lipolysis with glucose homeostasis. For example: despite being subject to severe diet-induced obesity and TG accumulation, ATGL-deficient mice have improved glycaemic control. This has been linked to an impaired ability to liberate free fatty acids (FFA) from adipose TG depots, and a resultant marked reduction in circulating fasting FFA. To compensate for this lack of circulating FFA to satisfy metabolic demand, there is an increased reliance on glucose, and as such, improved glucose clearance as measured by glucose- and insulin-tolerance tests [15]. Further highlighting the reliance of *Pnpla2*^*−/−*^ mice on glucose to satisfy metabolic demand is an increased respiratory quotient that deviates further from wild type mice during an 18 h fast. During fasting *Pnpla2*^−/−^ mice also gradually decrease energy expenditure, resulting in a significant reduction in VO_2_ as glucose and glycogen stores are exhausted. Thermal regulation is also impaired in *Pnpla2*^−/−^ mice as they are unable to maintain normal body temperature upon exposure to a cold (4 °C) environment 4 °C [15].

## ATGL-deficient mice are cold intolerant due to impaired cardiomyocyte fatty acid oxidation

While adipocyte TG storage in mice deficient in ATGL was significantly elevated, the magnitude of this effect was arguably smaller than many in the field had expected. In contrast, the defining alteration in lipid homeostasis in the total knockout was not the development of overt obesity, but rather an affliction with severe cardiac TG accumulation [15]. Interestingly, this phenotype is similar to the cardiomyocyte TG accumulation and cardiomyopathy seen in humans with mutations in the *PNPLA2* gene [16]. In mice, this phenotype resulted in premature mortality due to heart failure beginning by twelve weeks of age. Interestingly, mice that are heterozygous for ATGL deficiency (*Pnpla2*^*+/−*^) also displayed mild cardiac dysfunction, and studies have found that this can be rectified using cardiomyocyte-specific ATGL overexpression [17–19].

Although contractile failure in cardiac muscle was originally attributed to the gross TG over accumulation [15], it is the subsequent mitochondrial dysfunction that leads to cardiac failure in these mice [20]. Thus, the cardiac failure that afflicts ATGL-deficient mice is ultimately due to mitochondrial defects that prevent efficient mitochondrial substrate oxidation and respiration [20]. This effect is independent of substrate availability as the addition of exogenous glucose or palmitate does not rescue the cardiac defect in ATGL-deficient mice, rather it may be linked to ATGL-generated FFAs that activate the peroxisome proliferator-activated receptors (PPAR’s). Both monounsaturated (e.g. oleic acid) and polyunsaturated fatty acids (e.g. linoleic acid) are capable of activating PPARs, however other molecules such as fatty acid-Coenzyme A (CoA), eicosanoids, and phospholipids have also been shown to activate PPARs [21]. Accordingly, pharmacologic treatment of ATGL deficient mice with synthetic PPARα agonists reversed mitochondrial defects associated with ATGL deficiency, restored cardiac function, and significantly reduced cardiac TG accumulation reinforcing the notion that ATGL-generated PPAR ligands are necessary to sustain adequate mitochondrial function [20]. Furthermore, it is known that restoration of ATGL to adipose tissue but not cardiomyocytes is capable of providing ligands that stimulate PPAR-α, PPAR-δ, and PGC-1α, for example palmitoleic, oleic, and linoleic acid [22–26]. This suggests that the restoration of mitochondrial function is as result of ATGL-derived PPAR ligands rather than exogenously synthesized ligands.

Rather than being used directly as a fuel substrate, there is some evidence that exogenous FA must first be esterified into TG within the cell and then subjected to local lipolysis by ATGL before it can be utilized for oxidation [27]. This seemingly futile cycle of re-esterification of FA into TG may be a local control mechanism to protect the cell from dysregulated lipid metabolism [15, 28]. When exogenous FA are taken into a cell, they are esterified to fatty acid acyl CoA’s in the cytoplasm. Upon FA conversion into its esterified acyl CoA form it can be either directed to the mitochondria to undergo oxidation or directed to the endoplasmic reticulum to be incorporated into TG. Both FA and their esterified CoA form can act as detergents causing ER stress. Additionally, saturated fatty acids, such as palmitic acid are capable of inducing apoptosis and are not readily incorporated into TG whereas unsaturated fatty acids such as oleic acid are more readily incorporated into TG [21, 29]. The incorporation of excess FA into TG is also associated with decreased levels of other potent lipid signalling molecules such as ceramides and diglycerides. Oxidation of exogenous FA is reduced but not completely abolished in global ATGL knockout, and is not impaired in the brown adipose-specific ATGL deficient mouse. Thus, this cycle of re-esterification of FA into TG followed by subsequent lipolysis and oxidation may be a localized control mechanism to protect the cell from excess intracellular FA in periods of high FA flux into the cell rather than a prerequisite for FA oxidation. Additionally, by reducing intracellular FA levels there is also a significant reduction in other potent lipid signalling molecules such as ceramides and diglycerides [15, 28].

Interestingly, this detailed study of cardiomyocyte lipolysis revealed a surprising role for cardiac lipid metabolism in thermogenic regulation. ATGL-deficient mice are cold intolerant and this effect was initially attributed to the alteration of brown adipose tissue lipolysis (BAT), which caused it to gain a white adipose tissue (WAT)-like appearance [15] . However, the generation of mice lacking ATGL exclusively in BAT, through inducible manipulation of the *Pnpla2* gene either by diphtheria toxin-A using the UCP1 promoter [30] or by injection of tamoxifen in the inducible UCP1-*Cre* mouse model [31], revealed no associated impairments in thermogenesis. Instead, it revealed that the cold intolerance accompanying ATGL deficiency is due to inadequate fuel availability in cardiomyocytes, rather than defective BAT or WAT function, per se [30, 31]. BAT-specific ATGL deficiency renders mice unable to hydrolyse BAT TG, while WAT TG lipolysis remains uncompromised. As a result, circulating FFA, which are derived primarily from WAT lipolysis, are normal in BAT-specific *Pnpla2*^*−/−*^ mice, and these provide sufficient substrate to maintain thermoregulation upon cold exposure [31, 32]. It could still be argued, based on these findings, that impaired thermogenesis in *Pnpla2*^*−/−*^ mice is likely to be a result of impaired BAT-mediated fatty acid oxidation occurring secondary to reduced WAT FFA liberation. However, Schreiber and colleagues have demonstrated that cold tolerance is restored by cardiomyocyte-specific expression of ATGL in *Pnpla2*^*−/−*^ mice through the use of the α-MHC-ATGL transgene, where ATGL is deficient in both WAT and BAT [31]. This phenomenon has been confirmed in mice with tamoxifen-inducible cardiomyocyte ATGL ablation [17, 31]. Despite adequate circulating FFA, loss of cardiomyocyte ATGL causes the development of hypothermia upon cold exposure underscoring the importance of ATGL in sustaining cardiac function and thermoregulation.

## Adipose-specific knockout reveals compensatory mechanisms to reduce FFA storage

Improved insulin sensitivity and glucose tolerance in *Pnpla2*^*−/−*^ mice has been investigated using tissue-specific models. Given that ATGL was first discovered in adipose tissue, and is most highly expressed in adipose tissue, it is not surprising that there was significant interest in generating an adipose-tissue specific *Pnpla2*^−/−^ model. Several groups have independently generated adipose-specific ATGL-deficient mice with comparable results [28, 33, 34]. These results from these adipose-specific knockouts were achieved using different transgenic systems, specifically under the control of *aP2*-*Cre* [28], *adiponectin*-*Cre* [33], and *Fabp4*-*Cre* [34]. Similar to the global ATGL knockout, the adipose-specific models demonstrate impaired adipose TG lipolysis during 4 h [34] or 12 h fasts [35], increased TG accumulation in WAT and BAT, decreased cold tolerance during fasting, and improved systemic insulin sensitivity. These mice also show reduced circulating plasma FFA and TG. Deletion of ATGL in adipose tissue also results in reduced energy expenditure and impaired exercise performance in mice, which is attributed to a reduction in fuel availability [26, 35, 36]. Additionally, adipose-*Pnpla2*^*−/−*^ compensates to reduce FFA oxidation and TG storage, which may help to explain the smaller-than-expected elevation in adipose tissue TG content [15]. Specifically, these mice display reduced activity of PPAR-α and –γ target genes in WAT, BAT, and liver, including *Pgc1α*, *Acox1*, and *Cpt1α* [33]. In adipocytes, PPAR-γ is known to play a major role in TG storage, inducing increased expression of genes responsible for de novo *lipogenesis* and adipocyte differentiation [37]. Interestingly, adipose-specific deletion of ATGL resulted in reduced hepatic leucocyte infiltration and activation, specifically showing resistance to high-fat diet induced expression of *Mcp1* and *Tnfα* suggesting these mice are also protected from high-fat diet induced liver inflammation [33]. Additionally, in WAT, BAT, and liver the reduction in PPAR-α activity reduced expression of genes associated with β-oxidation, likely due to the decrease in FFA flux to these tissues accompanying the impaired ability of the adipose ATGL knockout to mobilize adipose TG [33, 37].

Converse to ATGL-deficiency, overexpression of ATGL in adipocytes using a transgene under the control of *aP2* prevented high-fat diet induced obesity and decreased TG levels in adipose tissue [38]. Surprisingly, despite the increase in adipose TG lipolysis ATGL-over expressing mice did not display an increase in fasting FFA. This result was attributed to two phenomena, both facilitated within the adipocyte: 1) Increased β-oxidation and 2) increased re-esterification cycling of DG to TG [38]. Another intriguing phenomenon noted in the ATGL-over expressing mouse was improved insulin sensitivity compared to controls [38]. This was surprising given that the global and adipose tissue knockouts displayed improved insulin sensitivity [15, 28, 33, 34]. The global- and adipose-knockouts displayed a significant reduction in fasting FFA [15, 28, 33] while the transgenic ATGL-overexpressing mice displayed normal fasting FFA [38]. In both of these models, in addition to reduced adipose TG, there was a marked reduction in hepatic steatosis accompanied by an increase in hepatic gluconeogenesis and peripheral uptake of glucose by skeletal muscle [33, 38]. Combining the observations of improved insulin sensitivity with reduced and normal fasting FFA levels in the knockout and transgenic over-expressing models, respectively, it indicates the improvement in insulin sensitivity in these models is not directly mediated by circulating levels of FFA from WAT. Instead this improvement in global insulin sensitivity is likely mediated, in part, by hepatic insulin signalling [33], which will be discussed below.

## ATGL-deficient mice have increased reliance on glucose in skeletal muscle

As the largest site of both glucose and fatty acid disposal, a role for skeletal muscle was investigated in some of the earliest studies using tissue-specific *Pnpla2* knockouts and, indeed, much of the improved insulin sensitivity noted in the global knockout has been attributed to an increase in glucose uptake by skeletal muscle amid decreased circulating FFA levels [39–41]. This improved insulin sensitivity also correlated with impaired mobilization of fat stores, forcing ATGL-deficient mice to have greater reliance on glucose and glycogen stores. In addition to increased glucose uptake by skeletal muscle, global ATGL-deficient mice display increased markers of insulin responsiveness, specifically increased phosphorylation of phosphatidylinositol 3-kinase (PI3K) and Akt in vivo in skeletal muscle [40]. One possibility for the improvement in insulin sensitivity may be due to a reduction in ligands that result in generation of by-products that influence insulin sensitivity (i.e. ceramides). It has been shown that FFA in particular can mediate ceramide production through binding to inflammatory receptors such as TLR4 [42–44]. Excess FFA has also been implicated in inducing skeletal muscle inflammation and insulin resistance in obese subjects through stimulating TLR4 and TLR2 [45]. In the context of ATGL deficiency we would except reduced skeletal muscle inflammation due to the decreased presence of ligands capable of signalling through TLR. However, when ATGL-deficient mice are infused with an excess supply of FFA they still exhibit a normal depletion of myocellular glycogen stores and glucose clearance [39]. A second possibility for the increase in glucose use by skeletal muscle is a reversal of the Randall effect, whereby due to the increased proportion of glucose compared to FFA there is decreased ability of skeletal muscle to oxidize FFA’s [46]. However skeletal muscle from ATGL deficient mice actually displays an improved capacity to acquire exogenous FFA and maintain a normal capacity to oxidize FFA [39]. Given these two observations it suggests that the increased use of glucose by skeletal muscle in ATGL-deficient mice is necessary to satisfy metabolic demand rather than biochemical preference or capability to oxidize a particular fuel.

Despite the central role of skeletal muscle as a sink for both glucose and fatty acids, deletion of ATGL exclusively in skeletal muscle, through the use of a *Myo-Cre* system, did not influence systemic insulin sensitivity [47]. Indeed, ATGL deficiency exclusively in skeletal muscle was accompanied by a robust increase in myocellular TG levels, however glucose clearance, response to insulin, and weight gain on a high fat diet was not different between skeletal muscle ATGL-deficient and wild type control mice [47]. ATGL-over-expression in skeletal muscle in vivo, through the use of a transgene under the control of the *Ckm* promoter, also did not alter measures of systemic glycaemic homeostasis despite intramyocellular decreases in TG. Interestingly, in vivo ATGL over-expression in mice does not result in accumulation of DG in skeletal muscle [47]. However over-expression of ATGL in primary human myotubes resulted in a marked increase in DG, subsequent activation of PKC, and disrupted insulin signalling [48, 49]. Furthermore, DG accumulation has been shown to induce insulin resistance in vivo, most notably in the global HSL-knockout [13]. This is in stark contrast to the ATGL global knockout, which does not accumulate DG [15], further highlighting the differential ability between TG and DG in induction of insulin resistance. In the context of ATGL- and HSL-deficient mice we would expect accumulation of DG within the cytoplasm rather than a membrane bound component within the cell. This subcellular compartmentalization may contribute to the absence of an effect of increased presence of DG upon ATGL over-expression in vivo, as demonstrated by targeted knockout down of CGI-58 where despite an accumulation of DG there was no chance in insulin sensitivity or signalling [50, 51]. The lack of DG accumulation despite ATGL over-expression in skeletal muscle in vivo is likely due to an ability to compensate for the increased lipolysis of TG to DG, by increasing DG catabolism to monoglyceride or re-esterification of DG to TG [47].

## Liver ATGL is required for normal liver function but not systemic glycaemic control

In contrast to initial reports that the liver does not express ATGL [15], recent reports found it expressed at low levels that are physiologically relevant for efficient hepatic lipid metabolism, since loss of hepatic ATGL causes hepatic steatosis [52, 53]. Interestingly, early reports indicated that in global ATGL deficient mice hepatic insulin signalling is impaired [40], however in adipose-specific ATGL deficient mice hepatic insulin signalling is improved [33]. This effect of improved insulin sensitivity in the adipose-specific knockout versus the global knockout suggests that the improvement in systemic insulin sensitivity of the global knockout is not entirely dependent on the liver. It also indicates the hepatic ATGL has an important role in insulin sensitivity of the liver. Indeed, there are multiple reports showing correlation of reduced hepatic ATGL, that are associated with increased hepatic steatosis and impaired liver function in non-alcoholic fatty liver disease [52–54]. Furthermore, in the adipose-specific ATGL knockout the liver helps compensate for the systemic reliance on glucose by increasing gluconeogenesis and reducing hepatic de novo *lipogenesis* [33]*.*

Mice with liver-specific ablation of ATGL, using the albumin-*Cre* promoter, are characterized by progressive development of hepatic steatosis that was exacerbated with age; accumulating 3-times more hepatic TG compared to a 2-fold increase of hepatic TG in the global knockout despite no change in overall body mass [15, 55]. Additionally, hepatic ATGL was not required to induce the improvement in systemic insulin sensitivity seen in the global- and adipose-specific ATGL knockouts, however hepatic ATGL is necessary for the improvement in hepatic insulin sensitivity [53, 55, 56]. Indeed, systemic glycaemic and lipid control remained similar between wild type and liver-specific ATGL deficient mice. Liver ATGL deficient mice had similar plasma glucose, FA, and TG levels compared to wild type mice after a 6 h fast and had similar performance in glucose and insulin tolerance tests [55]. This indicates that hepatic ATGL deficiency does not affect insulin sensitivity in extra-hepatic tissues.

Consistent with increased hepatic steatosis there were also large increases in markers of liver damage, including plasma alanine aminotransferase and aspartate aminotransferase. Interestingly, liver-specific ATGL deficiency resulted in significantly decreased expression of genes involved in FA oxidation, including PPARα. Hepatic very low-density lipoprotein (VLDL) secretion remained normal as fasting serum lipid levels remained similar to wild type controls and hepatic microsomal triglyceride transfer protein levels remained normal [55]. Conversely, adenoviral overexpression of hepatic ATGL was able to ameliorate hepatic steatosis in obese mice. This over-expression did not improve systemic glycaemic control despite improved markers of hepatic insulin sensitivity [54, 56]. Combining these observations, it indicates that hepatic ATGL is not required to induce the systemic improvement in insulin sensitivity, however it is important in hepatic lipid metabolism.

## β-cell ATGL is required for normal insulin secretion

Pancreatic β-cells are key regulators of systemic glycaemic control as one of their main functions is the production and secretion of insulin in response to feeding [57]. Much of the research into insulin resistance and metabolic syndromes has focused of tissues of nutrient storage (i.e. fat) and disposal (i.e. heart, muscle, and liver), however there is also evidence that β-cell dysfunction contributes to the progression of the metabolic syndrome [58–60]. Despite improved glycaemic control, global ATGL deficient mice have impaired glucose stimulated insulin secretion and ATGL-deficient mice display basal hypoinsulinemia [15, 61]. Furthermore, β-cells isolated from ATGL deficient mice display reduced insulin release ex vivo and ATGL inhibition by shRNA in vitro also results in impaired glucose stimulated insulin secretion [61].

Ablation of ATGL specifically in β-cells recapitulates in vitro findings of impaired glucose stimulated insulin secretion [25, 62]. However, there are two distinct models that employ β-cell-specific ATGL ablation that display contradictory findings in measures of metabolic control. We will briefly outline the findings between these two models. Tang et al. [25] reported that the metabolic phenotype accompanying β-cell ATGL deficiency is primarily due to decreased generation of PPARδ ligands. The reduced availability of PPARδ ligands causes mitochondrial dysfunction and impaired mitochondrial oxidation that is ultimately driving the impaired glucose stimulated insulin secretion. However, Attané et al. [62] reported normal mitochondrial function, consistent with their previous in vitro studies [61]. Attané and colleagues attribute impaired insulin control to reduced generation of monoacylglycerols that control insulin release [58, 59]. Additional contradictory findings include differences in glycaemic control. Specifically, Tang et al. found decreased glucose tolerance [25] while Attané found improved glycaemic control despite impaired insulin section [62] The major experimental differences between these two models are: mouse strain used (C57BL/6 J vs. C57BL/6 N), *Cre*-promoter gene used (*Rip-cre* vs. *Mip-cre*), and age of mice used (8 weeks vs. 23 weeks of age) for Tang et al. vs. Attané et al., respectively [25, 62]. An important aspect of these differences is the use of C57BL/6 J mice, which display a predisposition to glucose intolerance and impaired insulin secretion, due to a mitochondrial defect [63]. The mitochondrial defect in the C57BL/6 J background could be rectified by addition of PPARδ ligands, which under physiological conditions are generated locally by β-cell ATGL, that are not required with the normal mitochondrial activity in the C57BL/6 N background. This impairment in mitochondrial function could directly impact glucose oxidation capacity and subsequent glucose disposal. However, given the consistent findings of impaired insulin secretion, we can conclude that ATGL expressed in β-cells is implicated in the impaired insulin secretion seen in these models.

## Targeting ATGL pharmacologically

Initial interest in developing therapeutic agents manipulating ATGL in a clinical setting was halted upon the discovery of the severe cardiac defects and steatosis seen in the global knockout. Given the reversal of the cardiac-defect in the heart-sparing knockout [26] coupled with the improvement in insulin sensitivity and hepatic steatosis in the adipose-specific knockout there has been a resurgence of interest in targeting ATGL pharmacologically [28, 33, 34]. The ATGL inhibitor Atglistatin has been shown to improve insulin sensitivity and reduce weight gain in high-fat diet fed mice, while ameliorating the dramatic lipid accumulation seen in tissues of the global knockout [64, 65]. However, Atglistatin does not inhibit lipolysis in human adipocytes [66] but instead has off-target effects by inactivating carboxylesterases 1 and 2 [66, 67]. A thorough discussion of pharmacological inhibitors of lipases is beyond the scope of this review, however the reader is referred to the recent review by Quiroga and Lehner [67].

## Conclusion

**Fig. 1:**
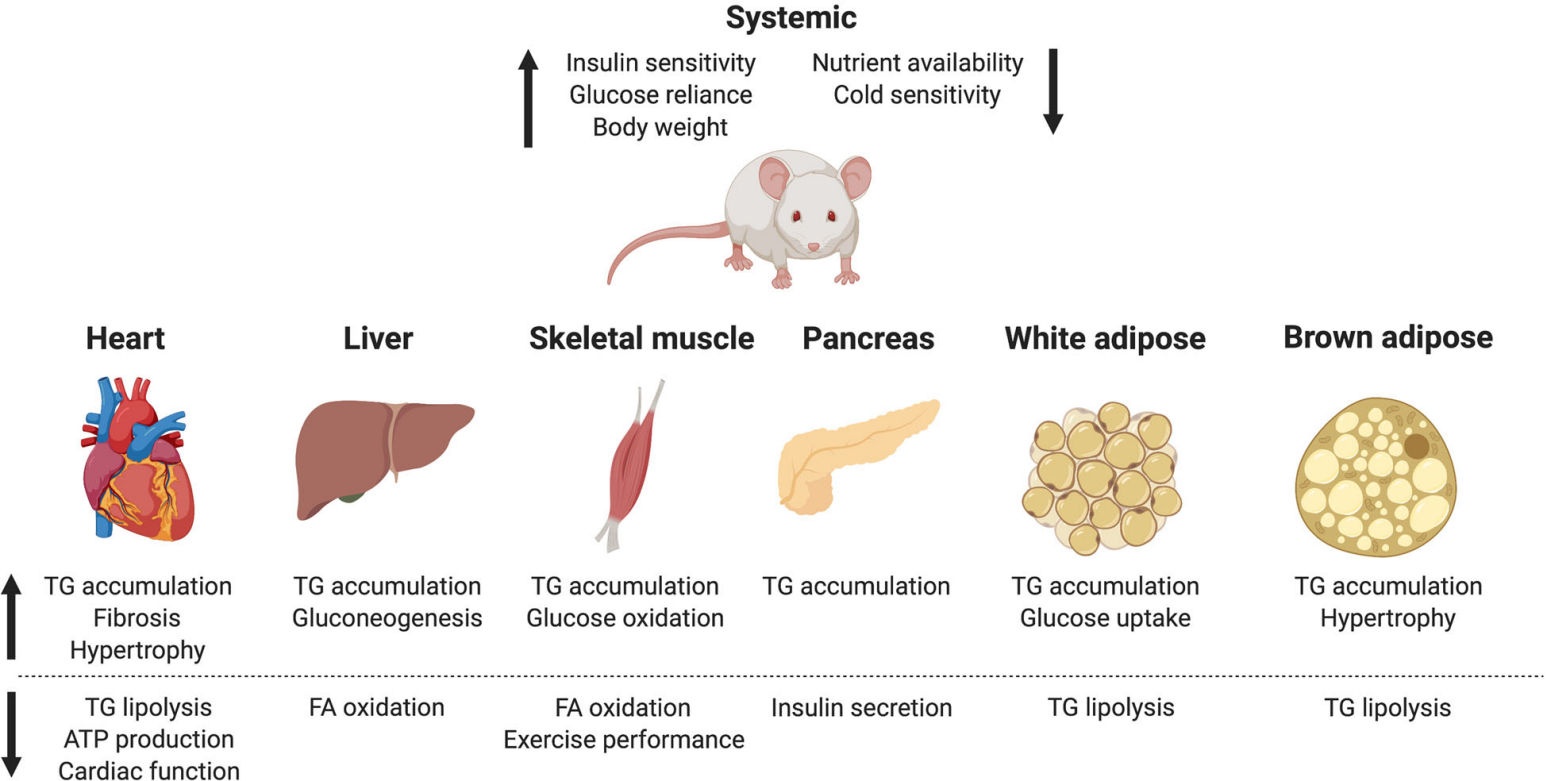
Summary of reported effects of global ATGL ablation in key tissues. These effects are summarized from reports by multiple groups with a common feature of localized TG accumulation and greater dependence on glucose metabolism. Created with BioRender.com

Since the initial characterization of ATGL as an adipose TG lipase in 2004, the use of transgenic mice has revealed important contributions of ATGL to systemic lipid and glucose homeostasis (summarized in Fig. 1) [11]. It is evident that although ATGL was initially characterized only in WAT, it has important tissue-specific roles in other tissues, including BAT, cardiac muscle, skeletal muscle, pancreas and liver, as summarized in Tables 1 and 2. In cardiomyocytes ATGL is required to prevent fatal cardiac TG accumulation. Adipose ATGL is critical in supplying the body with adequate substrates to satisfy metabolic demand in the form of FFA. Hepatic-ATGL is required to prevent hepatic steatosis and maintain normal liver function. Importantly, this review provides a unique perspective on the contributions of ATGL to insulin sensitivity that is independent of TG accumulation, which normally correlates with insulin resistant states [68, 69].

**Table 1:** Effects of ATGL deficiency in mice on lipid metabolism across various tissues compared to control littermates

Tissue of Interest					
Tissue-knockout	Liver	WAT	BAT	Heart	Skeletal Muscle
Global^a^	TG accumulation Decreased TG synthesis Decreased FFA oxidation	TG accumulation Decreased TG synthesis	TG accumulation Decreased TG synthesis No change in FFA oxidation	TG accumulation Decreased TG synthesis Decreased FFA oxidation	TG accumulation Decreased FFA oxidation
Liver^b^	TG accumulation Decreased TG synthesis Decreased FFA oxidation	No change	No change	No change	No change
Adipose^c^	Decreased fasting TG	TG accumulation Decreased TG synthesis	TG accumulation Decreased TG synthesis	No change	No change
BAT^d^	Unknown	No change	TG accumulation Decreased TG synthesis No change in oxidation	Unknown	No change
Heart^e^	Unknown	Unknown	Unknown	TG accumulation Decreased TG synthesis Decreased FFA oxidation	No Change
Skeletal muscle^f^	Unknown	Unknown	Unknown	Unknown	TG accumulation
Pancreas^g^	Unknown	Increased TG lipolysis	Increased FFA oxidation	Unknown	Unknown

^a^ key reference: [15], ^b^ key references: [55, 56], ^c^ key references: [28, 33, 34], ^d^ key references: [31, 32], ^e^ key references: [17–20], ^f^ key references: [36, 39, 47], ^g^ key references: [25, 61, 62]

**Table 2:** Effects of ATGL deficiency in mice on glucose metabolism across various tissues compared to control littermates

Tissue of Interest					
Tissue-knockout	Liver	WAT	BAT	Heart	Skeletal muscle
Global^a^	Increased gluconeogenesis Decreased fasting glycogen	Increased uptake Increased glucose oxidation	Increased glucose oxidation	Decreased glycolysis	Increased glucose oxidation
Liver^b^	No change	No change	No change	No change	No change
Adipose^c^	Increased gluconeogenesis Decreased glycogen levels	Increased glucose uptake	Increased glucose oxidation	Increased glucose oxidation	Increased glucose oxidation
BAT^d^	Unknown	Unknown	No change	Unknown	No change
Heart^e^	Unknown	Unknown	Unknown	Decreased glycolysis	No change
Skeletal muscle^f^	No change	No change	No change	No change	No change
Pancreas^g^	Unknown	Unknown	Unknown	Unknown	Unknown

^a^ key reference: [15], ^b^ key references: [55, 56], ^c^ key references: [28, 33, 34], ^d^ key references: [31, 32], ^e^ key references: [17–20], ^f^ key references: [36, 39, 47], ^g^ key references: [25, 61, 62]

## Data Availability

Not applicable.

## Abbreviations

ATGL: Adipose triglyceride lipase
BAT: Brown adipose tissue
DG: Diglyceride
FFA: Free fatty acid
HSL: Hormone-sensitive lipase
Pnpla2: Patatin like phospholipase domain containing 2
PPAR: Peroxisome proliferator-activated receptor
TG: Triglyceride
WAT: White adipose tissue
